# Simvastatin Blocks Blood-Brain Barrier Disruptions Induced by Elevated Cholesterol Both In Vivo and In Vitro

**DOI:** 10.1155/2012/109324

**Published:** 2012-02-01

**Authors:** Xijuan Jiang, Maojuan Guo, Jinling Su, Bin Lu, Dongming Ma, Ruifeng Zhang, Lin Yang, Qiang Wang, Yiwen Ma, Yingchang Fan

**Affiliations:** ^1^Department of Pathology, Tianjin University of Traditional Chinese Medicine, Tianjin 300193, China; ^2^Department of Experimental Teaching, Tianjin University of Traditional Chinese Medicine, Tianjin 300193, China

## Abstract

*Background*. Hypercholesterolemia and disruptions of the blood brain barrier (BBB) have been implicated as underlying mechanisms in the pathogenesis of Alzheimer's disease (AD). Simvastatin therapy may be of benefit in treating AD; however, its mechanism has not been yet fully understood. *Objective*. To explore whether simvastatin could block disruption of BBB induced by cholesterol both in vivo and in vitro. *Methods*. New Zealand rabbits were fed cholesterol-enriched diet with or without simvastatin. Total cholesterol of serum and brain was measured. BBB dysfunction was evaluated. To further test the results in vivo, rat brain microvascular endothelial cells (RBMECs) were stimulated with cholesterol in the presence/absence of simvastatin in vitro. BBB disruption was evaluated. *Results*. Simvastatin blocked cholesterol-rich diet induced leakage of Evan's blue dye. Cholesterol content in the serum was affected by simvastatin, but not brain cholesterol. Simvastatin blocked high-cholesterol medium-induced decrease in TEER and increase in transendothelial FITC-labeled BSA Passage in RBMECs. *Conclusions*. The present study firstly shows that simvastatin improves disturbed BBB function both in vivo and in vitro. Our data provide that simvastatin may be useful for attenuating disturbed BBB mediated by hypercholesterolemia.

## 1. Introduction

The blood-brain barrier (BBB), a barrier between the central nervous system (CNS) and the systemic circulation, maintains homeostasis within the brain microenvironment. The anatomical substrate of the BBB is the cerebral microvascular endothelium, which, together with astrocytes, pericytes, neurons, and the extracellular matrix, constitutes a “neurovascular unit” that is essential for the health and function of the CNS. Failure of the BBB is a critical event in the progression of several diseases, such as Alzheimer's disease (AD) [1–4]. BBB breakdown is accompanied by an increase in the trans-endothelial permeability to substances, which can damage the microenvironment of the brain and affect the structure and function of the CNS. Therefore, it is not surprising that strategies have been developed to “repair” the BBB in order to restore normal brain homeostasis and prevent the infiltration of pharmacologically active (noxious) substances into the brain. Cholesterol is one of the most notorious natural risk factors for arteriosclerotic cerebrovascular disease [5, 6]. Mounting evidence suggests that cholesterol also plays a critical role in the early stage of AD [7–9]. Hypercholesterolemia leads to increased BBB leakage, an effect that may contribute to AD pathogenesis [10, 11]. Thus, blocking BBB disruption may have beneficial effects against AD caused by hypercholesterolemia. Several epidemiological studies have revealed that cholesterol-lowering statins, which are used for the treatment of coronary arterial disease, are associated with a decreased risk of developing AD. However, the mechanisms underlying this effect remain unclear [8, 12]. Statins have been shown to ameliorate BBB dysfunction resulting from a number of conditions, including diabetes, transient focal cerebral ischemia, and HIV-1 [13–15]. However, the effects of statins on BBB disruptions induced by hypercholesterolemia have not been reported. To address this question, the permeability of the BBB was studied in vivo using rabbits fed a cholesterol-enriched diet and treated with simvastatin, a widely used natural statin derived from fermentation. The effect of simvastatin on BBB was also performed using rat brain microvascular endothelial cells (RBMECs) cultured under high-cholesterol conditions in vitro.

## 2. Materials and Methods

### 2.1. Chemicals

Simvastatin capsules used in vivo were purchased from Nantong Hua Pharmaceutical Co., Ltd (Jiangsu, China). The simvastatin used in vitro and Evans blue dye were obtained from Sigma (St. Louis, MO, USA). Dulbecco's Modified Eagle's Medium (DMEM), fetal bovine serum (FBS), streptomycin, penicillin, DNase I, collagenase, collagenase/dispase, and fluorescein-isothiocyanate-conjugated bovine serum albumin (FITC-BSA) were obtained from Invitrogen (Carlsbad, CA, USA). Antibodies to ZO-1 and occludin were purchased from Zymed (San Francisco, CA, USA).

### 2.2. Animals and Treatment

Twenty-four adult male New Zealand White rabbits (2356 ± 212 g) were used in this study. Rabbits were housed individually in the rabbit facility and were kept on a 12 h light/12 h dark cycle at 28 ± 2°C and 37–48% humidity. Animals were randomly assigned to three groups. The *control diet group* (*n* = 8) consisted of rabbits that received normal rabbit chow for 10 weeks. The other rabbits were fed chow supplemented with 2% cholesterol for 6 weeks and were then randomly assigned to either the *cholesterol group* (*n* = 8), which was fed chow supplemented with 2% cholesterol or the *cholesterol + simvastatin group* (*n* = 8), which was fed chow supplemented with 2% cholesterol and 5 mg/kg/d simvastatin for an additional 4 weeks. The olfactory bulbs, brain regions with an intact BBB under normal physiological conditions, have been implicated in Alzheimer's disease by studies reporting that AD patients experience olfactory dysfunction [16]. Other research has shown that the hippocampus is characterized by a selective fragility of the BBB and is often affected by AD pathology early in the disease process [17]. Accordingly, the olfactory bulbs and hippocampus were two primary brain areas examined in this study. All experiments were approved by the Committee for Animal Care and Use at Tianjin University of Traditional Chinese Medicine.

### 2.3. Evan's Blue Leakage Assay

To assay BBB permeability, Evans blue dye (4%; 25 mg/kg) was injected into rabbits through the ear vein and allowed to circulate for 3 h before the animals were anesthetized with pentobarbital sodium salt (30 mg/kg). While deeply anesthetized, animals were perfused with 37°C saline via the left cardiac ventricle to wash out any vascular Evans blue. Following perfusion, brains were quickly removed, and the olfactory bulbs and hippocampi were isolated, weighed, and incubated for 72 h with formamide in the dark at room temperature (25°C). After incubation, samples were centrifuged at 10,000 ×g for 10 min, supernatants were collected, and the absorbance was determined (Ex 620 nm and Em 680 nm) with a Jasco FP-777 spectrofluorimeter (Jasco UK Ltd, Essex, UK). Evan's blue concentrations were calculated from standard curves, and results were expressed as Evan's blue/specimen wet weight [18].

### 2.4. Immunohistochemistry

To evaluate the expression of the tight junction proteins, occludin and zonula occluden-1 (ZO-1), cryostat brain sections (10 *μ*m) of the olfactory bulbs and hippocampi were fixed with acetone and then rinsed 3 times in 0.01 M PBS for 5 min each. Fluorescently labeled primary antibodies against ZO-1 (Zymed, clone ZO1-1A12, dilution 1 : 200) and occludin (Zymed, clone OC-3F10, dilution 1 : 200) were diluted in PBS containing 1% Triton X-100, applied to the brain sections and incubated overnight at 4°C in a dark humidified chamber. Sections were rinsed 3 times in 0.01 M PBS for 5 min each and were subsequently examined by Leica microscopy. Images were analyzed with Image J software.

### 2.5. Serum and Brain Cholesterol Measurements

Total serum cholesterol was measured in venous blood collected from rabbit ear veins using standard enzymatic techniques with a Fully Automatic Biochemistry Analyzer. Following perfusion, brains were removed quickly and the cortex and hippocampus were removed and weighed. Cholesterol levels in the cortex and hippocampus were measured using reverse-phase HPLC (ZORBAX XDB C18 4.6 × 250 mm Spheri-5 RP C18 column, 5 *μ*m, Agilent, flow rate, 0.8 mL/min) with Varian Prostar 325 ultraviolet detection at 208 nm and LC Workstation V6.2 Chromatographic Data System.

### 2.6. Primary Culture and Treatment of Rat Brain Microvessel Endothelial Cells

Primary cultures of rat brain microvessel endothelial cells (RBMECs) were isolated from Wistar rat (approximately 100 g) brains using a combination of enzyme digestion and ultracentrifugation approaches as described previously [19], with minor modifications. In brief, fresh rat brains were obtained, and the surface vessels and meninges of the brains were removed. The grey matter was cut into 1 mm^3^ pieces, digested with 39 U/mL DNase I and 0.7 mg/mL collagenase at 37°C for 1 h, and centrifuged at 1000 ×g for 10 min. After centrifugation, the supernatant was discarded, 20% BSA was added, and the resulting mixture was centrifuged at 1000 ×g for 20 min. The dark red pellet was collected and subjected to further enzymatic digestion in 39 U/mL DNase I and 1 mg/mL collagenase/dispase for 1 h. After centrifugation, the supernatant was discarded, and brain capillary fragments were seeded onto matrigel-coated Transwell filters (a pore size of 0.4 *μ*m Transwell, Corning Life Sciences, USA) in Dulbecco's modified Eagle's medium supplemented with 20% FBS, 100 *μ*g/mL heparin, 30 *μ*g/mL ECGF, and 2 mM glutamine. The purity of RBMECs was >95% as determined by factor-VIII-related antigen and immunocytochemical staining (data not shown). These cultures, which were used as an in vitro model of the BBB, were maintained for 8–10 days to obtain confluence prior to starting experiments. When a stable transendothelial electrical resistance **(**TEER) value was obtained, the cultures were washed twice with serum-free DMEM and treated with water-soluble cholesterol (10 *μ*M) for 24 h with or without simvastatin (5 *μ*M) to evaluate disruption transendothelial permeability [20].

### 2.7. Transendothelial Electrical Resistance (TEER) Measurement with RBMECs

TEER was measured using a Millicell-ERS electrical resistance apparatus (Millipore, Eschborn, Germany) 24 h after treatment with simvastatin. To study the effects of simvastatin on TEER, the Transwell inserts with the RBMECs cultures mentioned above were placed into the chamber electrode filled with HEPES-buffered (25 mM) serum-free DMEM [21]. The symmetrically apposing electrodes were situated above and beneath the membrane, allowing a uniform current density to flow across the membrane. The resistance was recorded when the meter indicated a stable resistance. Resistance values of multiple transwell inserts from each experimental group were expressed in common units (Ω/cm^2^) after subtracting the value of a blank cell-free filter.

### 2.8. Measurement of Transendothelial Protein Passage

Transendothelial permeability to macromolecules was assessed by passage of fluorescein isothiocyanate-conjugated bovine serum albumin (FITC-BSA) across the monolayer as described previously [22]. In brief, primary RBMECs were treated with cholesterol in the presence/absence of simvastatin as mentioned above. FITC-BSA (50 *μ*g/mL) was added to the upper chambers and its diffusion across the model BBB was monitored using an FL600 microplate fluorescent reader (Biotek, Winooski, VT) (Ex 488 nm, Em 525 nm) 3 h after the addition of FITC-BSA. BBB permeability was calculated using the following formula: (BSA lower chamber) × 100/(BSA upper chamber).

### 2.9. Statistical Analyses

All data were expressed as mean ± SD. Statistical significance for multiple comparisons was determined using one-way ANOVAs and Tukey's post hoc tests with SPSS software (SPSS, Chicago, IL, USA). The significance threshold was set at *P* < 0.05.

## 3. Results

### 3.1. Simvastatin Attenuates High Cholesterol Diet-Induced Leakage of Evan's Blue Dye In Vivo

**Figure 1:**
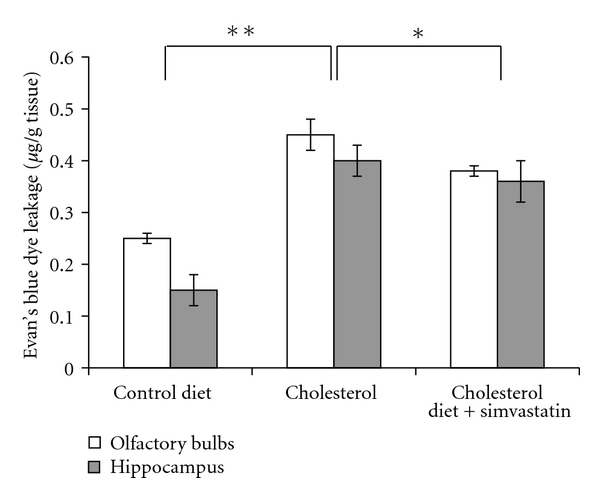
Simvastatin blocks high-cholesterol diet-induced leakage of Evan's blue dye. New Zealand White rabbits were fed a normal diet (10 weeks) or a high-cholesterol diet (10 weeks) with or without simvasatin (over the final 4 weeks). After treatment, BBB permeability was evaluated by measuring Evan's blue dye leakage. High-cholesterol diet significantly increased the leakage of Evan's blue dye into the olfactory bulbs and hippocampus, and these effects were attenuated by treatment with simvastatin (5 mg/kg/day during weeks 7–10) (*n* = 8, *P < 0.05; **P < 0.01).

BBB integrity was assessed by Evans blue extravasation (Figure 1). Evans blue is normally excluded from the brain parenchyma by the BBB and is only detectable when the integrity of the BBB is compromised. Increased BBB permeability was observed in rabbits fed a high-cholesterol diet (supplemented with 2% cholesterol for 10 weeks), as evidenced by the increased Evans blue content in their olfactory bulbs and hippocampi when compared to those of rabbits fed a normal diet. Oral administration of simvastatin at a dose of 5 mg/kg/day for 4 weeks (weeks 7–10) attenuated the high cholesterol diet-induced leakage of Evan's blue dye in both the olfactory bulb and the hippocampus.

### 3.2. Simvastatin Had No Effect on the High-Cholesterol Diet-Induced Downregulation of Tight Junction Proteins In Vivo

**Figure 2:**
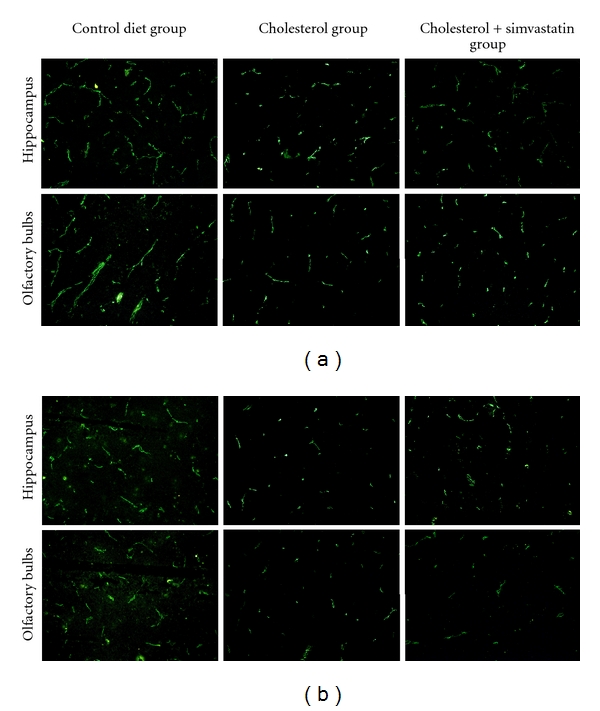
Simvastatin has no effect on high-cholesterol diet-induced downregulation of tight junction proteins. Cryostat sections of the olfactory bulbs and hippocampus were incubated with fluorescently labeled antibodies against occludin and ZO-1, and representative images for each treatment group are shown. (a) Decreased ZO-1 immunostaining was observed in the olfactory bulbs and hippocampus of cholesterol-fed rabbits (supplemented with 2% cholesterol for 10 weeks). This effect was not blocked by treatment with simvastatin (5 mg/kg/day for 4 weeks). (b) Decreased occludin immunostaining was observed both in the olfactory bulbs and hippocampus of cholesterol-fed rabbits, and this effect was not blocked by simvastatin (200×).

**Figure 3:**
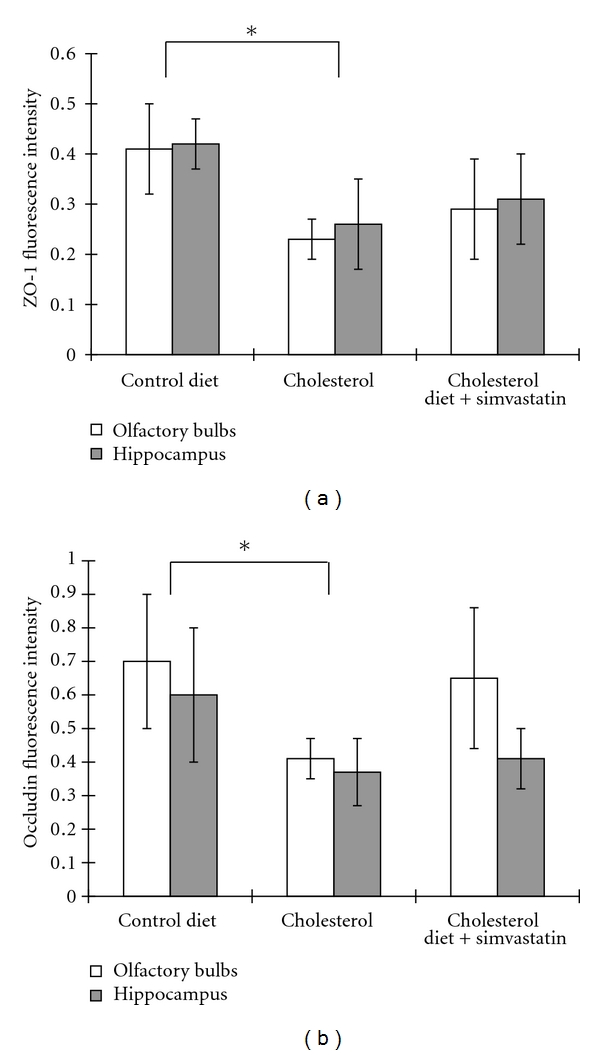
Simvastatin has no effect on high-cholesterol diet-induced downregulation of tight junction proteins. Images from Figure 2 were analyzed with Image J software. (a) Quantitative data from Figure 2(a) demonstrates that a high-cholesterol diet significantly decreases ZO-1 immunoreactivity in the olfactory bulbs and hippocampus, an effect that was not blocked by simvastatin. (b) Quantitative data from Figure 2(b) demonstrates that a high-cholesterol diet significantly decreased occludin immunoreactivity in the olfactory bulbs and hippocampus. However, this effect was not blocked by simvastatin treatment (5 mg/kg/day for 4 weeks) (*n* = 8, *P < 0.05).

The tight junctions (TJs) between the endothelial cells serve to restrict blood-borne substances from entering the brain. ZO-1 and occluding were the major interendothelial junctional proteins. Thus, to determine if the effects of simvastatin on BBB permeability resulted from alterations in the major interendothelial junctional proteins, the expression of ZO-1 and occludin was examined in rabbits fed a high-cholesterol diet treated with simvastatin. The results revealed decreased immunostaining for ZO-1 (Figures 2(a) and 3(a)) and occludin (Figures 2(b) and 3(b)) in the olfactory bulbs and hippocampi of rabbits fed a high-cholesterol diet for 10 weeks when compared to rabbits fed a normal diet. However, simvastatin (5 mg/kg/day for 4 weeks) had no effects on either the expression of occludin or ZO-1.

### 3.3. Simvastatin Affects Plasma Levels of Cholesterol but Not Levels of Cholesterol in the Brain

Cholesterol seems to play an important role in the development of AD. Therefore, it is of interest to evaluate the effects of high doses of simvastatin on levels of cholesterol in serum and in brain. As expected, rabbits fed a cholesterol-enriched diet for 10 weeks exhibited over a 20-fold increase in total serum cholesterol concentration. Treatment of high-cholesterol-diet-fed rabbits with simvastatin (5 mg/kg/day for 4 weeks) significantly reduced plasma cholesterol levels. However, cholesterol levels in the hippocampus and cortex were not affected by a cholesterol-enriched diet in the presence or absence of simvastatin (Table 1).

### 3.4. Simvastatin Blocks the TEER Decline and Increased Transendothelial Protein Permeability Induced by High Levels of Cholesterol In Vitro

**Figure 4:**
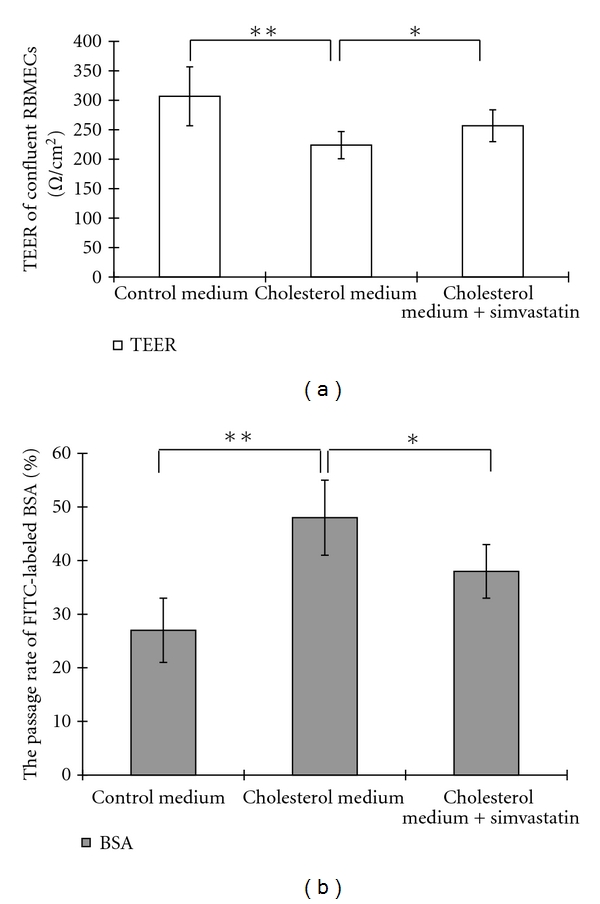
Simvastatin blocks cholesterol-induced TEER decline and transendothelial permeability in vitro. Rat primary brain endothelial cells were stimulated for 24 h with 10 *μ*M cholesterol in the presence or absence of simvastatin (5 *μ*M). TEER of confluent RBMECs (a) and permeability of FITC-BSA (b) were measured. Simvastatin significantly blocks both the TEER decline and the increase in FITC-BSA permeability induced by high cholesterol (a, b). TEER was expressed in ohms per square centimeter for electrical resistance (*n* = 8, *P < 0.05; **P < 0.01).

To further verify the observed effects of simvastatin, primary cultures of brain endothelial cells, the most important component of the BBB, were examined. Primary RBMECs were stimulated with cholesterol (10 *μ*M) in the presence/absence of simvastatin (5 *μ*M) for 24 h. Potential changes in the integrity of the BBB were assessed by measuring TEER and the permeability of an RBMEC monolayer to FITC-BSA.  Figure 4(a) shows that cholesterol markedly retarded the development of TEER of RBMEC monolayers during postconfluent growth. A significant effect of simvastatin (5 *μ*M) became visible after a 24 h treatment of RBMECs. Consistent with the results of TEER, cholesterol was shown to induce increased permeability to the large molecular weight tracer FITC-BSA, and this effect was significantly blocked by simvastatin (Figure 4(b)).

## 4. Discussion

Our results showed that simvastatin significantly reduces leakage of Evan's blue dye across the BBB but does not affect the expressions of the tight junction proteins, occlud, and ZO-1, in rabbits fed a cholesterol-rich diet. In addition, our data indicate that simvastatin alters plasma levels of cholesterol but does not affect levels of cholesterol in the brain. We also observed that cotreatment with simvastatin blocked the cholesterol-induced increase in transendothelial permeability and the cholesterol-induced decrease in TEER observed in primary cultured RBMECs. The semipermeable BBB restricts the diffusion of blood-borne substances into the brain parenchyma. Disruptions in the BBB can cause chemical imbalances in the neuronal “milieu,” ultimately resulting in synaptic and neuronal dysfunction. Disruption of the BBB is a hallmark of Alzheimer's disease [10, 11]. Furthermore, elevated plasma cholesterol has been shown to increase BBB permeability and is a possible risk factor for AD [2, 23–26]. Consistent with the above findings, the present study demonstrated that high levels of cholesterol can induce leakage of the BBB in vivo and disrupt the integrity of in vitro models of the BBB.

AD, a type of dementia that affects millions of elderly individuals, is the fourth leading cause of death among the elderly in developed countries [27]. Current therapeutic interventions for AD are largely ineffective. Epidemiological studies reveal a lower prevalence of AD in patients with hypercholesterolemia who are taking statins, a frequently prescribed class of lipid-lowering drugs [28, 29]. Several experiments have shown that statins may be potentially beneficial in the treatment of AD via their direct effects on brain cholesterol metabolism [30, 31]. However, other studies have failed to demonstrate a reduction in the ratio between 24S-hydroxycholesterol and cholesterol, in response to statin treatment [32]. At the same time, recent retrospective epidemiological studies have reported that the use of statins, but not nonstatin lipid-lowering agents, may reduce the risk of developing AD [8, 12]. Consistent with previous findings [33], our results also demonstrate that short-term simvasatin treatment does not affect brain total cholesterol; however, it has an effect on BBB integrity. This result suggests that the mechanisms by which statins protect against AD may extend beyond their lipid-lowering effects. Amyloid deposition is seen as the core pathological aspects of AD. Cholesterol has been mainly considered for dementia through promoting the generation of amyloid. However, immunization trials targeting the removal of amyloid-*β* plaques in Alzheimer's disease have so far failed to stop the progression of dementia. Recent data show that to reduce oxidative stress and inflammation-related factors in the brain by interfering with the permeability of the BBB may enhance the therapeutic effect mentioned above [34]. Therefore, to maintain the integrity of the BBB maybe more closely associated with the action of simvastatin on preventing AD instead of lipid-lowering effects.

The current study provides the first evidence for the protective effects of simvastatin against BBB disruptions both in rabbits with hypercholesterolemia and in RBMECs exposed to cholesterol-enriched media. In rabbits fed a high-cholesterol diet, simvastatin reduced the leakage of Evan's blue dye into the olfactory bulb and hippocampus. To follow up these findings, we subsequently carried out an in vitro study using brain microvascular endothelial cells exposed to 10 *μ*M cholesterol as a model of BBB disruption. We found that simvastatin blocks the TEER decline and increased transendothelial permeability induced by high cholesterol, a result that corresponded well with our observed results in vivo. Because tight junctions between cerebral microvascular endothelial cells form the basis of the BBB [35], we examined the expression of two tight junction proteins, occludin and ZO-1, after simvastatin treatment. However, we found that simvasatin had no effect on the expression of tight junction proteins. These results suggest that simvastatin blocks BBB leakage induced by elevated cholesterol, independent of changes in the expression of the tight junction molecules, occludin and zonula occluden-1. This finding is in agreement with at least one previous report in the literature [22], which showed similar results in an in vitro model of multiple sclerosis.

In the present study, we did not explore the detailed molecular mechanisms whereby simvastatin protects against BBB disruption. However, we did demonstrate that simvastatin is effective in ameliorating BBB disruption induced by high-cholesterol diet in rabbits and in reducing cholesterol-induced endothelial permeability in an in vitro model of BBB disruption.

## 5. Conclusion

To the best of our knowledge, the present study is the first to show that simvastatin improves BBB integrity both in rabbits fed a high-cholesterol diet and in a cholesterol-induced in vitro model of BBB disruption using primary cultured RBMECs. Our data provide a new mechanism underlying the neuroprotective activity of simvastatin and suggest that simvastatin may be useful in the treatment of BBB disruptions induced by hypercholesterolemia.

## Figures and Tables

**Table 1:** Concentration of total cholesterol (TC) in the serum, cortex, and hippocampus of rabbits fed a control diet, high-cholesterol diet, and high-cholesterol diet plus simvastatin.

Group	Serum TC	Cortex TC	Hippocampus TC
Control diet	51.4 ± 9.96^b^	1.29 ± 0.16	1.36 ± 0.14
Cholesterol diet	1449.6 ± 677.1	1.25 ± 0.13	1.31 ± 0.23
Cholesterol diet + simvastatin	866.1 ± 147.46^a^	1.27 ± 0.11	1.38 ± 0.10

The concentration of total serum cholesterol is expressed as g/mL and the concentration of total cortex or hippocampus cholesterol is expressed as g/100 g wet weight of tissue. Data are expressed as means ± S.D. (*n* = 8;  ^a^ *P* < 0.05;  ^b^ *P* < 0.01; compared with Cholesterol diet).
